# A High-Frequency and Real-Time Ground Remote Sensing System for Obtaining Water Quality Based on a Micro Hyper-Spectrometer

**DOI:** 10.3390/s24061833

**Published:** 2024-03-13

**Authors:** Yunfei Li, Yanhu Fu, Ziyue Lang, Fuhong Cai

**Affiliations:** 1 School of Biomedical Engineering, Hainan University, Sanya 572000, China; 21110810000014@hainanu.edu.cn (Y.L.); fuyanhuxing@gmail.com (Y.F.); 2 School of Food Science and Engineering, Hainan University, Haikou 570228, China; 21210832000013@hainanu.edu.cn

**Keywords:** hyperspectral, water quality monitoring, inversion model, machine learning

## Abstract

The safeguarding of scarce water resources is critically dependent on continuous water quality monitoring. Traditional methods like satellite imagery and automated underwater observation have limitations in cost-efficiency and frequency. Addressing these challenges, a ground-based remote sensing system for the high-frequency, real-time monitoring of water parameters has been developed. This system is encased in a durable stainless-steel shell, suited for outdoor environments, and features a compact hyperspectral instrument with a 4 nm spectral resolution covering a 350–950 nm wavelength range. In addition, it also integrates solar power, Wi-Fi, and microcomputers, enabling the autonomous long-term monitoring of water quality. Positioned on a rotating platform near the shore, this setup allows the spectrometer to quickly capture the reflective spectrum of water within 3 s. To assess its effectiveness, an empirical method correlated the reflective spectrum with the actual chlorophyll a(Chla) concentration. Machine learning algorithms were also used to analyze the spectrum’s relationship with key water quality indicators like total phosphorus (TP), total nitrogen (TN), and chemical oxygen demand (COD). Results indicate that the band ratio algorithm accurately determines Chla concentration (R-squared = 0.95; RMSD = 0.06 mg/L). For TP, TN, and COD, support vector machine (SVM) and linear models were highly effective, yielding R-squared values of 0.93, 0.92, and 0.88, respectively. This innovative hyperspectral water quality monitoring system is both practical and reliable, offering a new solution for effective water quality assessment.

## 1. Introduction

Inland water, crucial for both human survival and ecosystem balance, possesses an inherent self-restoration ability. Although it can recover from pollution through natural processes, human activities such as overconsumption and industrial waste discharge disrupt this balance. The subsequent damage to the self-repair system leads to nitrogen and phosphorus over-enrichment, spurring rampant aquatic plant growth, particularly that of algae [1,2]. This algal bloom depletes dissolved oxygen, endangering other aquatic life and disrupting the food chain. Sustainable water resource management necessitates rigorous monitoring of water’s chemical and physical properties, understanding and controlling water quality parameters, and managing human interactions with inland water systems. Chlorophyll a (Chla), indicative of algal abundance, is a vital water quality parameter [3,4]. It is prevalent in algae, cyanobacteria, and other aquatic plants, providing a measure for eutrophication. Nitrogen and phosphorus, principal nutrients for these organisms, when excessive, trigger algal blooms, exacerbating eutrophication [5]. This proliferation heightens the Chla concentration, worsening water quality. Consequently, it is crucial to monitor not only Chla but also nitrogen and phosphorus content. This comprehensive understanding of nutrient status enables the development of effective strategies to prevent and control eutrophication.

Water quality monitoring usually involves manual sampling, utilizing the chemical properties of substances in water to measure the specific content of each parameter. This method is called in situ measurement. Although in situ measurement is the most accurate technique in evaluating water quality, its data collection process is often time-consuming, dangerous, expensive, and labor-intensive, coupled with inconsistent data measurements from different organizations and a lack of spatial and temporal continuity, making in-site measurement less applicable in many cases. With the development of remote sensing technology, remote sensing has become a supplement to traditional methods for monitoring aquatic ecosystems due to its advantages such as easy access, long-term dynamic monitoring, and low cost. Remote sensing water quality monitoring utilizes the fact that different components in the water selectively absorb or backscatter electromagnetic waves from different light sources. By receiving the intensity of electromagnetic waves reflected from the water surface through remote sensing devices, the content of components in the water can be quantitatively analyzed [6]. Researchers use satellites such as MODIS, TM, Sentinel-3, and ETM+ to remotely sense water bodies, and then extract reflection spectra and spatial information to explore the temporal and spatial distribution of Chla in water bodies [7,8,9]. Hyperspectral technology is widely used in fields such as remote sensing reconnaissance, agricultural monitoring, environmental science, and medical diagnosis by capturing the reflected or emitted spectra of an object at different wavelengths and realizing detailed analyses of the composition and properties of materials [10,11,12]. Researchers use hyperspectral remote sensing data to predict and evaluate other indicators in water, such as concentrations of heavy metals [13]. There are also scientific researchers who carry out regression between suspended sediment concentration, conductivity, total suspended solid (TSS) concentration, and water spectra [14,15]. There are some challenges in obtaining water quality spectra for inversion through remote sensing. Firstly, we need to consider the obstruction of vegetation and atmospheric interference, which will have a certain impact on water quality monitoring [16,17]. Vegetation can block light from entering water bodies, and aerosols, gases, and clouds in the atmosphere can also interfere with the propagation and reflection of light, making it relatively difficult to accurately obtain the reflection spectrum of water bodies [11,18,19,20]. Secondly, the reflection signal of water bodies is usually weak, especially in the visible light band. Approximately 80% of the energy received by sensors comes from the reflection of the atmosphere and sunlight, while the reflection signal from water bodies only accounts for a small portion. This makes accurately extracting water quality information from remote sensing data even more challenging [21]. In addition, using remote sensing technology for water quality monitoring requires expensive sensors and satellite equipment, and is limited by satellite task arrangements and data acquisition, making it difficult to achieve long-term and real-time water quality monitoring.

In recent years, some scientific researchers have begun to explore the use of unmanned aerial vehicles equipped with spectrometers to obtain spectral information on water bodies [22,23,24], in order to overcome problems such as the low spatial resolution of satellite remote sensing images and interference from cloud and mist in water reflection spectra. Drones have higher spatial resolutions and can provide more detailed water information. However, using drones for spectral flight requires a certain cost, and calibration is required before and after the flight to ensure data accuracy. Drone remote sensing still cannot solve the problem of the mirror reflection energy generated by the sun shining on the water surface—the dazzling light entering the sensor. In addition, there are already several systems that monitor the water quality of inland waters in real-time through wireless sensor networks [25]. However, the underwater detectors of these systems are also susceptible to water erosion and pollution, which can be effectively addressed through ground-based remote sensing systems [26,27].

There is an urgent need to develop an effective alternative method to monitor key water quality parameters in inland waters, including chlorophyll, nitrogen, and phosphorus. For this purpose, a new type of high-frequency real-time ground remote sensing system has been specially designed. The system features a 350–900 nm spectrometer that can capture the spectra of the sky and water in just 3 s. The system relies on solar power and integrates wireless networks and microcomputers, enabling the independent long-term monitoring of water quality. In addition, a regression model has been developed to predict water quality by examining the relationship between spectral data and various water quality parameters. This provides a sustainable and low-maintenance solution for continuous environmental monitoring.

## 2. Materials and Methods

### 2.1. Autonomous Solar-Powered Micro-Hyperspectral Water Quality Monitoring System

**Figure 1:**
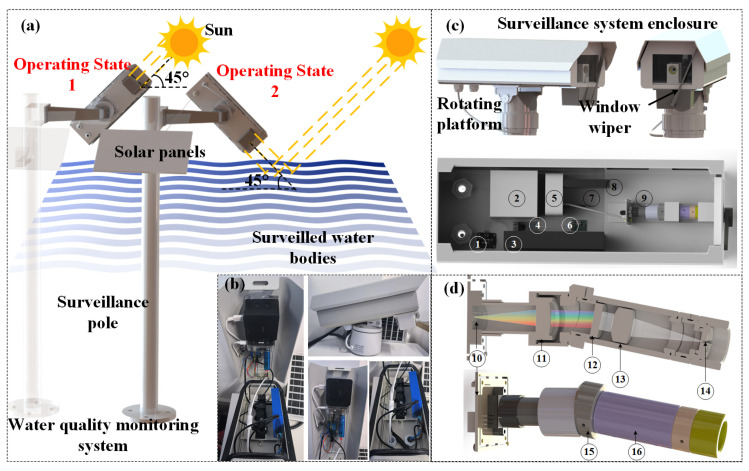
(**a**) Schematic diagram of the water quality monitoring system; (**b**) photo of the monitor; (**c**) 3D model of the monitor. The monitor is composed of the following components internally: 1. electricity monitoring board; 2: microcomputer; 3: battery; 4: 12 V to 5 V transformer; 5: USB expansion dock; 6: 12 V to 24 V transformer; 7: TTL to USB converter; 8: Wi-Fi; 9: microspectrometer. (**d**) Optical design of one spectrometer. The spectrometer system comprised elements below 10: a camera with a CMOS (IMX174, Sony, Tokyo, Japan); 11: doublet lens; 12: grating; 13: doublet lens; 14: slit; 15: 10°angled optic mounts (SM1L03T, Thorlabs, Newton, NJ, USA); 16: Lens Tube.

Figure 1a shows the configuration and working process of the water quality monitoring system. It includes a surveillance pole, solar panels, and a monitor. Figure 1b is a physical image of the monitor, and Figure 1c is a three-dimensional model of the monitor. The monitor consists of three parts: a surveillance system enclosure, a rotating platform, and a window wiper. The surveillance system enclosure is a stainless-steel box used to hold internal instruments, and the rotating platform allows the monitor body to rotate. The monitor is installed on the survey pole and rotates through the rotating platform. During the operation process, the monitor initially rotates to operation state 1 to capture the spectrum of the current sunlight at a 45° angle relative to the incident sunlight. This captured spectrum is denoted as $R_{sun}$. Subsequently, driven by the rotating platform, the monitor clockwise rotates to operating state 2, where it forms a 45° angle with the monitored water body. In this state, the monitor records the current spectrum of the water body, denoted as $R_{water}$. To obtain the current diffuse reflectance spectrum, R, of the water body, the spectrum of the monitored water body, $R_{object}$, is divided by the solar spectrum (R), as shown in Formula (1):

(1) $$R_{object}=\frac{R_{water}}{R_{sun}}$$

Figure 1c shows the internal electronic and communication circuits of the monitor system, as well as the microspectrometer and power supply. In order to improve the operational reliability of the system under different sunlight conditions, the high-efficiency battery equipped inside the system can collect and store energy when there is sufficient sunlight for use when there is insufficient sunlight, ensuring the stable operation of the system even in areas with significant changes in sunlight. The solar panel’s electricity is stored in a 12 V power supply using the electricity monitoring board. This board monitors the battery’s storage capacity. If the battery level drops below 10%, the circuit between the solar panel and the power supply is connected, enabling the battery to store solar energy. Conversely, if the battery level exceeds 95%, the circuit between the solar panel and the power supply is automatically disconnected to prevent overcharging and potential damage to the battery. During operation, power is supplied to the microcomputer and USB docking station via a 12 V to 5 V transformer. The CMOS (complementary metal-oxide semiconductor) at the end of the miniature spectrometer is connected to the computer through a USB, allowing the microcomputer to access the current image captured by the CMOS. This captured image represents the current spectral image, which will be further discussed in the following text. The expansion dock is equipped with a USB port that supports Wi-Fi connectivity. The microcomputer utilizes Wi-Fi to communicate with the outside world, enabling access to local area networks or obtaining spectral information on the currently monitored water body through mobile devices. Another interface on the expansion dock features a TTL to USB converter. This converter is utilized to control the windows wiper located at the front end of the monitor. A microcomputer program is responsible for scheduling the operation of the windows wiper, ensuring that it cleans any dust or water droplets present on the monitor’s front end. These elements can interfere with the accuracy and signal-to-noise ratio of the monitor’s spectrum acquisition. The power supply section is also connected to a 12 V to 24 V transformer, running parallel to the previously mentioned transformer. Its primary purpose is to provide power to the rotating platform. The rotating platform communicates with the microcomputer through USB and changes its working state by rotating under the control of the microcomputer. In summary, the mentioned components and functionalities form an integrated system that allows the monitor to capture and analyze spectral information of the monitored water body, communicate wirelessly, control the window wiper, and manage the rotating platform for different working states.

Figure 1d shows a schematic diagram of the spectrometer. Its optical components are installed in two lens tubes, which are connected through a 10° angle optical bracket (SM1L03T, Thorlabs, Newton, NJ, USA) to be compatible with the diffraction angle. Light enters through a slit in the front end and is collimated by a double lens. Then, it is diffracted with a grating of 300 lines/mm. Finally, the diffracted light is focused onto the CMOS chip through a second double lens.

### 2.2. Calibration of Hyperspectral Systems

**Figure 2:**
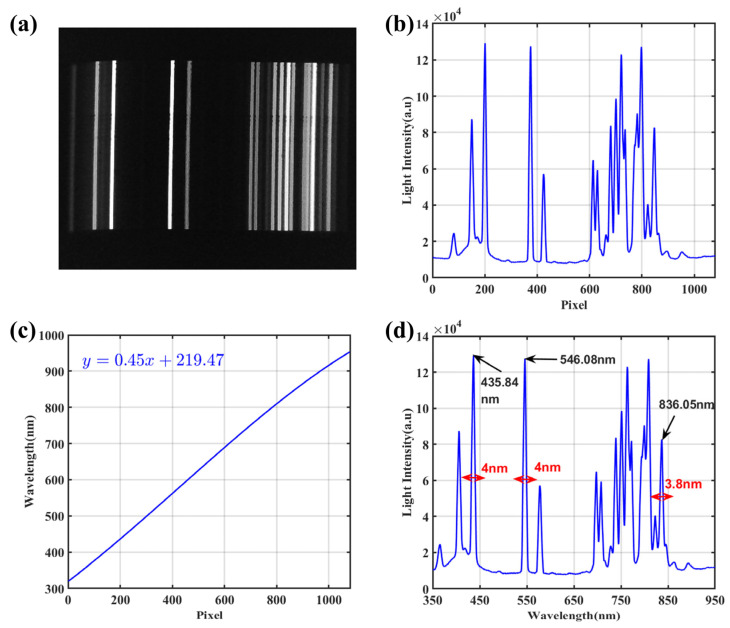
Calibration of the system using a mercury lamp: (**a**) spectra of the uncalibrated mercury lamp; (**b**) intensity of the spectra of the uncalibrated mercury lamp; (**c**) linear equation obtained from spectral calibration; (**d**) spectra of the mercury lamp after calibration.

A mercury lamp was used as a standard light source for spectral calibration. We calculate the spectral dispersion range of the imaging light passing through the grating by calibrating the system. The lamp’s spectrum had intensity peaks at wavelengths of 404.66 nm, 435.84 nm, 546.08 nm, 696.54 nm, and 738.40 nm, and their corresponding pixel indexes were 149, 200, 374, 612, and 679, respectively, as shown in Figure 2a. These experimental data are used to calculate the multi-order polynomial Equation (1) parameters, where λ represents the actual wavelength and x represents the pixel position. The grating diffraction equation is Equation (2). The first-order (*k* = 1) diffraction angle for 500 nm is 4.3 degrees. When the diffraction angle is small, sin*Θ* ≈ *Θ* and the relationship between the diffraction angle and wavelength is almost linear. Therefore, the values of *a*_2_ and *a*_3_ are close to zero.

(2) $$\lambda =a_{0}+a_{1}x+a_{2}x^{2}+a_{3}x^{3}$$

(3) 2dsinΘ=kλ

By bringing $a_{2}=a_{3}=0$ into Equation (1), the reconstructed parameters of $a_{0},a_{1}$ were 219.47 and 0.45, respectively. Figure 2c shows the relationship between the pixel index and the calibrated wavelength. The detection spectrum of the calibrated mercury lamp is shown in Figure 2d, and our spectrometer has a detection range of 350 nm to 950 nm. The spectral resolution of the system was also analyzed based on the full width at half maxima (FWHM) of the spectral peaks. As shown in Figure 2d, the FWHM of peaks at 546.08 nm and 836.05 nm are 4.0 nm and 3.8 nm, respectively. Therefore, the spectral resolution was about 3 nm~5 nm in the wavelength range between 350 nm and 950 nm.

### 2.3. Chlorophyll Fluorescence Regression in Water Bodies

In order to establish an inversion model for the chlorophyll concentration and reflectance spectra of water bodies, an experiment was conducted on a lake located in Hainan, China (18.73 N; 109.17 E). The abundance of algae in the current environment is reflected by the Chla in the water, which exhibits fluorescence characteristics. The commercial chlorophyll detector (RMD-ISY-10, Raimondotto, Beijing, China) was used to detect the standard value of a sample, and was read by placing the device in the water and waiting for 3 s. The commercially available RMD-ISY-10 is capable of emitting a 405 nm blue laser light to irradiate chlorophyll in water, which produced fluorescence, which was recorded using a built-in detector. The chlorophyll concentration was estimated based on the intensity of the chlorophyll fluorescence recorded using the detector. Subsequently, pure water was diluted at a ratio of 0.5. After each dilution, the chlorophyll detector was used to measure the Chla concentration, while a monitor was used to capture the spectra of the water body [28].

During the calculation of spectra, the spectra of the current water body were obtained by acquiring the sunlight spectra. Then, the reflection spectra of the water body were calculated. Empirical methods are commonly employed in the inversion model of Chla and reflectance spectra. Researchers calculate the correlation between water quality parameters and corresponding remote sensing reflectance, identifying characteristic bands or band combinations [29,30]. Statistical analysis, including regression models, is then used to develop an inversion algorithm between the water quality parameters and the selected characteristic bands or band combinations.

Overall, this experimental approach aimed to establish a relationship between chlorophyll concentration and the reflectance spectra of water bodies, using empirical methods and statistical analysis to derive an inversion model.

### 2.4. Regression of Nitrogen and Phosphorus Content in Water Bodies

The nitrogen and phosphorus content in water is crucial for assessing water body eutrophication. However, traditional spectral analysis methods face challenges due to the lack of distinct peaks for nitrogen and phosphorus that are within the visible range. To overcome this, machine learning is increasingly used for the statistical inversion of nitrogen and phosphorus content. Machine learning learns patterns and correlations by training spectral data and corresponding nitrogen and phosphorus content. Common techniques include support vector machine, artificial neural network, decision tree, and random forest [31]. These methods predict nitrogen and phosphorus content based on spectral characteristics. Machine learning handles complex relationships and achieves high prediction accuracy. By selecting and processing spectral data and optimizing training, machine learning compensates for the limitations of traditional methods, enhancing accuracy and reliability in nitrogen and phosphorus content inversion.

A lake located in Hainan, China (18.73 N; 109.17 E), served as the test subject for the inversion of the parameters nitrogen, phosphorus, and COD (chemical oxygen demand) using spectra and water quality data. The experiment involved obtaining water samples from the lake and performing equal-gradient dilution on the water quality. After each dilution, spectral extraction was conducted. In addition, an additional 10 mL of each sample was collected and stored for the detection of true concentrations. A commercial water quality tester (GL900, Green, Heze, Shandong, China) was used to measure the concentrations of the three water quality parameters: TP, TN, and COD in the samples. The process of total nitrogen (TN) determination includes three main steps: sample digestion, color reaction, and photometric measurement [32]. In the specific operation, the water sample is first mixed with potassium persulfate, and the nitrogen is converted into nitrate via high-temperature and high-pressure treatment; then, ascorbic acid and molybdate are added to trigger the reaction to produce a blue complex; finally, the absorbance is measured spectrophotometrically at a wavelength of 700 nanometers, and the TN concentration is calculated with reference to the standard curve. Total phosphorus (TP) and chemical oxygen demand (COD) were determined in a similar way [33,34].

## 3. Results

### 3.1. Inversion of Chlorophyll in Water Quality

**Figure 3:**
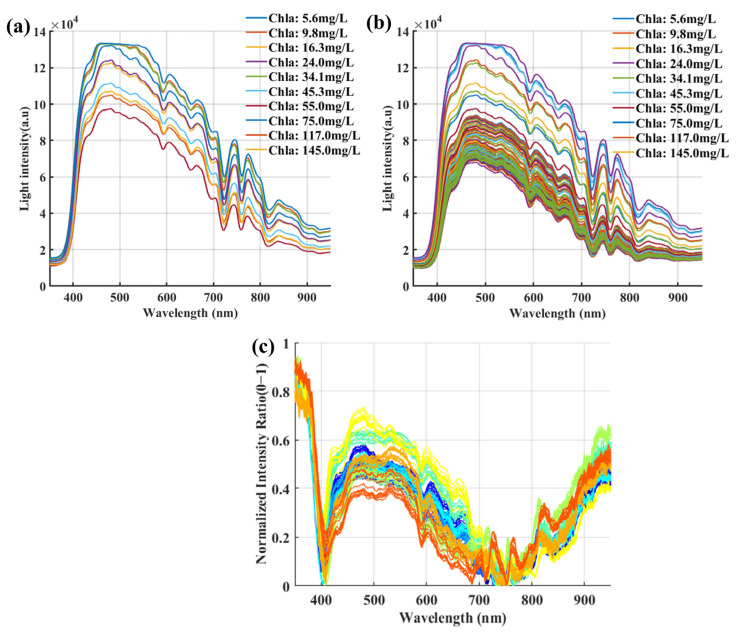
(**a**) Obtention of solar spectra at different chlorophyll concentrations. (**b**) Captured spectra of water samples with varying chlorophyll concentrations. (**c**) Measured reflectance spectra of water samples with different chlorophyll concentrations.

Figure 3 shows the sunlight spectra, the different concentrations, and water reflection spectra during the measurement process. Due to its absorption characteristics, the total reflection intensity of the water body does not exceed 1. In the wavelength range of 400–500 nm, as the wavelength increases, the water absorption decreases and the reflectivity increases. After 500 nm, the re-reflection characteristics are mainly affected by phytoplankton pigments. There is an obvious reflection peak near the green belt, indicating the presence of Chla. In the range of 680–750 nm, there is an absorption trough, which is a characteristic band of Chla concentration inversion. At longer wavelengths, the reflection effect of suspended matter becomes more significant, resulting in a significant reflection peak after 800 nm.

The industry widely acknowledges and utilizes algorithms such as the band ratio (red/near-infrared), reflection depth index, maximum chlorophyll index (MERIS), and three-band algorithms for assessing chlorophyll a concentration due to their capability to accurately delineate the relationship between water’s optical properties and chlorophyll content, which is crucial for water quality monitoring and environmental management. Building on this consensus, this study conducted an in-depth examination and application of these models to enhance the precision of chlorophyll a concentration estimation in water bodies, particularly in complex Type II waters. For instance, the band ratio algorithm selectively employs wavelengths around 670 nm and 700 nm to capture spectral features associated with chlorophyll a concentration. Moreover, given the strong correlation between the reflectance peak near 705 nm and chlorophyll a concentration, this research opted for 700 nm, 650 nm, and 750 nm as characteristic wavelengths for the three-band algorithm.

**Figure 4:**
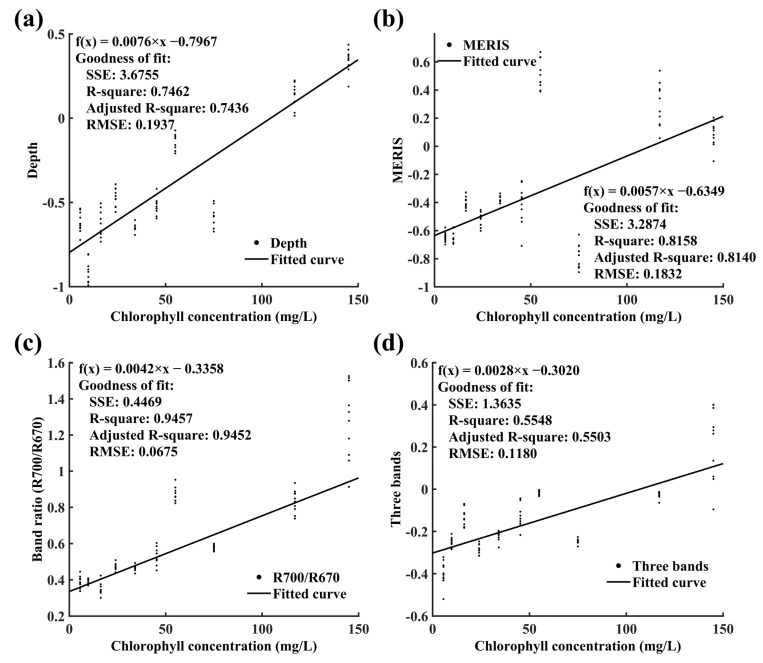
Chlorophyll spectra and spectral regression models established using different empirical models: (**a**) trough depth index, T-depth; (**b**) maximum chlorophyll index; (**c**) band ratio (near red/red); (**d**) three-band model.

Comparative analysis through Figure 4 demonstrates varying accuracies among the models in inverting chlorophyll a concentration. Figure 4a illustrates that the reflection depth index model, with an R-squared value of 0.7462, shows a significant correlation with chlorophyll a concentration but has room for improvement compared with other models. The maximum chlorophyll index model (MERIS), as depicted in Figure 4b, achieves an R-squared value of 0.8158 and an RMSE of 0.1832, indicating its high reliability in predicting chlorophyll a concentration in water bodies. Notably, the band ratio model (red/near-infrared), shown in Figure 4c, outperforms all compared models, with the highest R-squared value of 0.9458 and the lowest RMSE of 0.0675, underscoring its superior performance and prediction accuracy. Despite the lowest R-squared value of 0.5594 in Figure 4d, the three-band model holds significant value in analyzing the relationship between specific wavelength reflectance properties and chlorophyll a concentration, albeit with some limitations under certain conditions. 

### 3.2. Inversion of Water Quality Parameters: Nitrogen, Phosphorus, and COD

Currently, deep learning has demonstrated extensive capabilities in data analysis, especially in capturing intricate features and relationships within large datasets. For example, machine learning algorithms are used for target identification, analyzing the spectra of underwater minerals [35]. In water quality research, deep learning can extract features sensitive to variations in water quality parameters from hyperspectral data. This enables the accurate estimation of key indicators such as Total phosphorus (TP), total nitrogen (TN), and chemical oxygen demand (COD) in water bodies by learning the complex relationships between these features and water quality parameters. 

Two-hundred water quality samples of different concentrations were obtained via isogradient dilution. The reflectance spectra of these samples were extracted using the spectral system of this paper. The obtained spectral data were divided into the training set and test set in a ratio of 5:1, and a series of regression models, such as SOM, CNN, ANN, KNN, SVM, ET, RF, decision tree, partial least squares (PLS), and linear regression, were sequentially applied to invert the TP, TN, and COD parameters using the models. The models were evaluated on the basis of metrics such as R-squared, mean absolute error (MAE), root mean square error (RMSE) and mean absolute precision error (MAPE).

**Figure 5:**
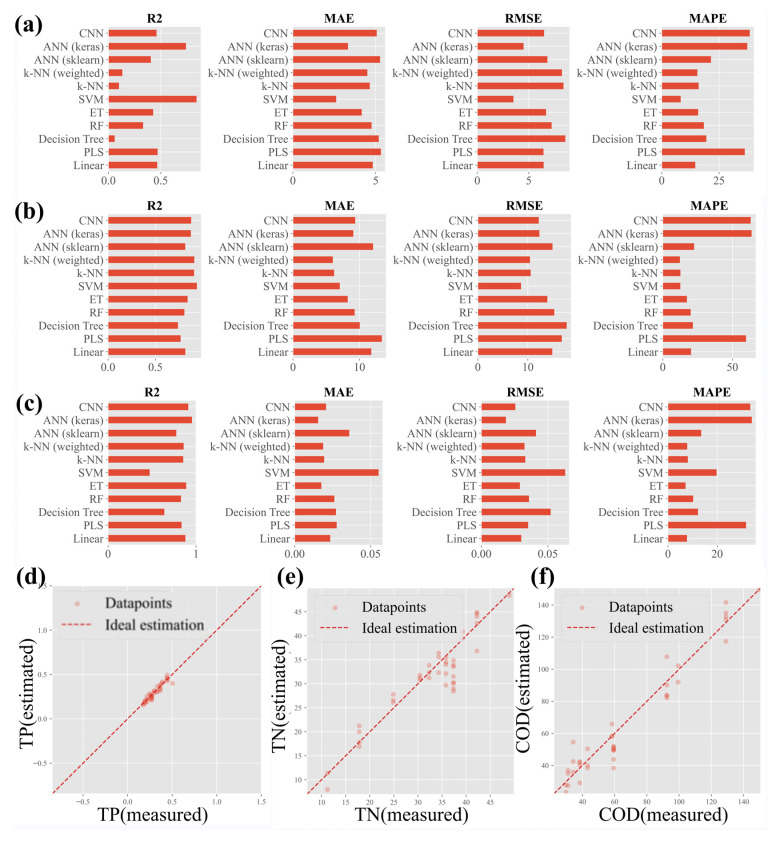
Comparison of regression model performance for hyperspectral data: (**a**) total nitrogen inversion model performance; (**b**) total phosphorus inversion model performance; (**c**) chemical oxygen demand (COD) inversion model performance; (**d**) scatterplot of total nitrogen prediction accuracy; (**e**) scatterplot of total phosphorus prediction accuracy; (**f**) scatterplot of COD prediction accuracy.

Figure 5 provides a succinct evaluation of regression models for TN, TP, and COD inversion. Figure 5a highlights the ANN (Keras) and SVM models’ superior R-squared values, indicating their robust fit to the observed data and substantial explanatory power. The accompanying lower MAE and RMSE values point to their predictive precision. Figure 5b showcases the SVM model’s adeptness in TP estimation, evidenced by a high R-squared of 0.85 and low error metrics. Figure 5c features the ANN (Keras) model’s exceptional fit for COD, with an R-squared of 0.93. Scatter plots in Figure 5d–f demonstrate the models’ accuracy, with data points closely aligned with the ideal prediction line, particularly for TN, TP, and COD inversions. This analysis attests to the integration of hyperspectral data and machine learning as a potent tool for water quality monitoring.

## 4. Discussion

The results of the study confirm the effectiveness and reliability of the newly proposed high-frequency real-time ground-based remote sensing system for water quality monitoring. The system consists of a compact hyperspectral instrument with a wide spectral range (350–950 nm) and fine resolution (4 nm), which is capable of assessing water quality parameters for the purpose of water quality monitoring. For chlorophyll regression modeling, the band ratio (R670/R700) model was the most effective, with an R-squared value of 0.95 and a root mean square error (RMSE) of 0.06 mg/L. Additionally, the support vector machine (SVM) and linear models showed excellent performance in predicting TP, TN, and COD with R-squared values of 0.85, 0.88, and 0.93, respectively. The mean absolute errors of these parameters were 0.02 mg/L, 2.62 mg/L, and 7.04 mg/L, respectively, and the corresponding root mean square errors were 0.03 mg/L, 3.51 mg/L, and 8.65 mg/L. The accuracy of these models was 91.7%, 92.3%, and 87.4%, respectively, which illustrates their robustness in water quality estimation.

These results emphasize the possibility of obtaining key water quality parameters by analyzing the reflectance spectra of water. In addition, the autonomous functional design of the system is supported by solar power and wireless connectivity, allowing for consistent, high-frequency monitoring. This pioneering ground-based remote sensing system offers great promise for the efficient and proactive management of water resources.

## Data Availability

The data supporting the results presented in this article are available upon request.
